# Which salivary components can differentiate metabolic obesity?

**DOI:** 10.1371/journal.pone.0235358

**Published:** 2020-06-29

**Authors:** Lucyna Ostrowska, Agnieszka Gornowicz, Barbara Pietraszewska, Krzysztof Bielawski, Anna Bielawska

**Affiliations:** 1 Department of Dietetics and Clinical Nutrition, Medical University of Bialystok, Bialystok, Poland; 2 Department of Biotechnology, Medical University of Bialystok, Bialystok, Poland; 3 Department of Synthesis and Technology of Drugs, Medical University of Bialystok, Bialystok, Poland; San Raffaele Roma Open University, ITALY

## Abstract

**Background:**

Obesity is a multifactorial disease and represents a global and relevant health problem. The aim of the study was to assess the concentration of pro-inflammatory cytokines (tumor necrosis factor-α (TNF-α), interleukin-8 (IL-8)) and other selected proteins as well as enzymes (soluble intercellular adhesion molecule 1 (sICAM1), calprotectin, matrix metalloproteinase-9 (MMP-9), matrix metalloproteinase-2 (MMP-2), toll like receptor 2 (TLR2)) detectable in the saliva of women who varied in body composition. It was debated whether there are marker factors in saliva that could indicate metabolic obesity.

**Methods and findings:**

The pilot study included 10 women with obesity (BMI>30 kg/m2) and 6 women with normal body weight (control group). The levels of TNF-α, IL-8, sICAM1, calprotectin, MMP-9, MMP-2, and TLR2 were checked by using the ELISA technique. We proved that women with metabolic obesity had significantly increased concentrations of IL-8, calprotectin, and MMP-2 in comparison with healthy subjects. Significant positive correlations of BMI with TNF-α, IL-8, and MMP-2 were observed. Similarly, the content of fat (in kg and %) in the bodies of the women correlated positively with TNF-α, IL-8, and MMP-2. Whereas, the visceral adipose tissue (VAT) correlated positively only with TNF-α and MMP-2, similarly to VAT/SAT. The WHR (waist hip ratio) was also positively correlated with TNF-α and MMP-2. Interestingly, we found that the level of insulin positively correlated with TNF- α concentration, which additionally confirmed metabolic obesity.

**Conclusions:**

We found that positive correlations of body mass index were observed only with salivary concentrations of TNF-α, MMP-2, and IL-8. Thus, it is worth conducting a study among a larger number of people taking into account these three salivary components.

## Introduction

Obesity is a chronic disease defined as excessive fat accumulation, which is a health risk. It is diagnosed when body mass index (BMI) (calculated as weight[kg]/height^2^[m^2^]) is equal to or more than 30 kg/m^2^. In 2018, the World Health Organization (WHO) estimated that in 2016 13% of adults aged 18 years or over were obese (over 650 million adults). Women suffered from this disease more often than men (15% of women vs. 11% of men) [1]. A special type of obesity is abdominal (visceral, central) obesity (occurring more commonly in men than women and increasing in both men and women with age) which is reported as “waist to hip ratio” or recognized when waist circumference exceeds 94 cm in men and 80 cm in women (for the European population). Central obesity is positively correlated with elevated fasting glucose concentrations, hypertension, dyslipidemia, and heart diseases [2].

Saliva consists of more than 400 types of proteins, where we can include: α-amylase, albumin, cystatins, histatins, lactoferrin, lysozyme, mucins, statherins, and transferrin. All these components possess different biological functions. A tremendous amount of salivary proteins demonstrate anti-viral, anti-fungal, and anti-microbial properties [3,4,5,6,7]. Leptin is also a component of saliva and its role is associated with the promotion of wound healing [8,9]. Many salivary factors are engaged in some diseases and might be useful tools for diagnosing some illnesses [10]. An increased concentration of proinflammatory cytokines, MUC5B, MUC1, histatin-5, and lactoperoxidase was demonstrated in the saliva of adolescents with dental caries [11,12,13]. Salivary biochemical markers, which are characteristic for periodontal disease, include enzymes (MMP-1 (matrix metalloproteinase-1), MMP-8 (matrix metalloproteinase-8), MMP-9 (matrix metalloproteinase-9), immunoglobulins, and a group of proteins (albumin, fibronectin). However, changes in the levels of salivary components is not only connected with oral diseases but might be useful as biomarkers for many others.

The aim of the study was to assess the concentrations of proinflammatory cytokines (TNF-α (tumor necrosis factor-α), IL-8 (Interleukin-8)), and other selected proteins as well as enzymes detectable in the saliva of women who varied in body composition. It was debated whether there are marker factors in the saliva that could indicate metabolic obesity. The correlations between selected anthropometric parameters and cytokines and other tested protein concentrations in the saliva of the examined women were also determined. We wanted to ascertain whether metabolic obesity differs from non-metabolic obesity in terms of the saliva components that indicate inflammation and to assess if it is worth doing research on a larger population (more saliva components were considered).

## Methods and materials

### Patients

The pilot study included 10 women with obesity (BMI>30 kg/m2) and 6 women with normal body weight (control group). The study was approved by the Medical University of Bialystok Bioethical Commission No. R-I-002/442/2015. We obtained written informed consent from the subjects. The subjects came to the Department of Nutrition and Clinical Nutrition to get nutritional advice. All subjects were weighed (Radwag) and measured, and then the individual BMI was calculated. The waist and hip circumferences were calculated (and the WHR (waist hip ratio). Body composition (fat content in kg and %, water content and basal metabolic rate—BMR) was measured and fat tissue content was scanned at the level of the umbilicus by bioimpedance using BioScan 920–2. During the same visit, saliva was collected to determine selected proinflammatory cytokines and other proteins.

### Salivary sample collection

Saliva was collected using a standard method. Samples from the subjects were collected between 9:00 and 11:00 a.m. All subjects abstained from eating and drinking for 2 h. The subjects rinsed their mouths with deionized water and were sitting in a comfortable position with their eyes open and head titled slightly forward. Unstimulated whole saliva was collected for 10 min by spitting, described by Navazesh [14]. Saliva samples were homogenized and clarified by centrifugation at 1200 RPMI for 15 min at 4°C. The aliquots of clarified supernatants were stored at –70°C for the mucins measurements.

### Determination of TNF-α, IL-8, sICAM1, calprotectin, MMP-9, MMP-2, TLR2

Highly sensitive assay kits (EIAab) were used to determine the concentrations of proteins in the salivary samples from the control and the experimental subjects. The microtiter plate provided in this kit was pre-coated with antigen-specific antibodies. Standards and samples were added to the appropriate microtiter plate wells. After two hours of incubation at 37°C, the plate was incubated with biotin-conjugated antibody for one hour at 37°C. Then, the microplate wells were aspirated and washed three times and then incubated with avidin conjugated to horseradish peroxidase (HRP). Next, a TMB (3,3',5,5'-tetramethylbenzidine) substrate solution was added to each well. The enzyme-substrate reaction was terminated by the addition of a sulfuric acid solution and the color change was measured spectrophotometrically 450 nm ± 2 nm. The antigen concentration in the samples was determined by comparing the O.D. to the standard curve.

### Statistical analysis

Statistical analysis was performed using Statistica 13.0 software from StatSoft. Medians and quartiles (Q1, Q3) were used. Interquartile range (IQR) was determined (as the difference between 1 and Q3). The Mann-Whitney U test and Spearman’s rank correlations were used to determine significant differences between the control and the study group (p < 0.05). HOMA-IR (Homeostatic Model Assessment—Insulin Resistance) was calculated from the formula, serum insulin concentration mU/ml x glucose concentration mmol/l / 22,5.

## Results

The selected proinflammatory cytokines and other proteins in the saliva were collected from 10 obese (49.2±11.08 years old) and 6 normal weight women (47.1±13.63). The characteristics of the study and the control group are shown in Table 1. Statistical analysis revealed that there were statistically significant differences between both groups in BMI, waist circumference, WHR, fat, visceral adipose tissue (VAT) (cm^3^, %), subcutaneous adipose tissue (SAT) (%), and the VAT/SAT ratio.

**Table 1:** Characteristics of the study and control group.

	Study group (n = 10)		Control group (n = 6)		p*
	Median	Q1-Q3	Median	Q1-Q3	
Age [years]	48.50	40.00–57.00	53.00	32.00–59.00	0.875
Weight [kg]	90.50	88.00–94.00	65.45	59.10–69.80	<0.001*
Height [m]	1.64	1.58–1.67	1.69	1.60–1.78	0.492
BMI [kg/m^2^]	33.93	31.91–36.36.06	22.84	22.54–23.09	<0.001*
Waist circumference [cm]	110.00	103.00–114.00	82.10	76.10–95.00	<0.001*
Hip circumference [cm]	117.00	116.00–118.00	98.00	96.10–100.00	<0.001*
WHR	0.94	0.88–0.98	0.84	0.79–0.92	0.042*
Fat [kg]	39.85	34.86–45.82	17.06	16.66–17.56	<0.001*
Fat [%]	44.74	39.17–47.77	28.38	24.47–29.49	<0.001*
VAT [cm^3^]	239.5	106.00–359.50	78.50	66.00–120.00	0.016*
SAT [cm^3^]	103	87.00–117.00	86.50	76.00–107.00	0.147
VAT [%]	65.39	54.83–77.71	47.97	45.46–52.03	<0.001*
SAT [%]	34.61	22.39–45.17	53.61	50.48–54.23	<0.001*
VAT/SAT	2.11	1.21–3.47	0.93	0.83–1.08	<0.001*

p*—statistically significant level <0.05. Comparison of women from the study and the control group (Mann-Whitney U test); BMI—body mass index, WHR—waist hip ratio, VAT—visceral adipose tissue, SAT—subcutaneous adipose tissue.

Subsequently, the concentrations of proinflammatory cytokines, such as TNF-α (tumor necrosis factor-α) and IL-8 (interleukin-8), in the saliva samples were determined and the results are shown in Table 2.

**Table 2:** Concentrations of pro-inflammatory cytokines and other tested proteins and enzymes in the salivary samples from the study and the control subjects.

	Study group (n = 10)		Control group (n = 6)		p*
	Median	Q1-Q3	Median	Q1-Q3	
**TNF-α [pg/ml]**	27.08	11.02–44.27	13.01	11.00–15.25	0.181
**Interleukin-8 [pg/ml]**	375.50	245.00–439.00	146.00	125.00–224.00	0.042*
**sICAM1/CD24 [pg/ml]**	15.46	11.60–56.30	16.05	8.61–17.36	0.368
**Calprotectin [pg/ml]**	98.40	47.80–160.90	35.10	17.00–45.00	0.042*
**MMP-9 [pg/ml]**	12.30	12.00–12.63	12.47	12.00–12.63	0.635
**MMP-2 [pg/ml]**	0.76	0.68–0.88	0.41	0.35–0.45	0.005*
**TLR2 [pg/ml]**	0.81	0.56–5.36	1.03	0.80–1.50	1.000

p*—statistically significant level <0.05. Comparison of the women from the study and the control group. (Mann-Whitney U test); TNF-α–tumor necrosis factor-α, sICAM1/CD24—soluble intercellular adhesion molecule 1, MMP-2 –metalloproteinase-2, MMP-9 –metalloproteinase-9, TLR2 –toll like receptor 2.

Median TNF-α saliva concentration in the study group was 27.08 (IQR: 33.07) pg/ml and the level was higher compared with the control group [13.01 pg/ml (IQR: 4.25)]. The difference between the groups was not statistically significant (p = 0.181.) (Table 2).

The concentration of interleukin-8 (IL-8) in the salivary samples from the study group ranged from 245 pg/ml (Q1) to 439 pg/ml (Q3) with a median of 375.5 pg/ml. The value was higher compared with the control group (p = 0.042), where it was 146 [IQR: 99]. (Table 2).

Median soluble intercellular adhesion molecule 1 (sICAM1) concentration in the salivary samples from the study group was 15.46 pg/ml. Whereas, in the control group the median was 16.05 pg/ml. The difference was not statistically significant (p = 0.368) (Table 2).

The level of calprotectin was also measured. The calprotectin calprotection in the salivary samples from the study group ranged from 47.80 pg/ml (Q1) to 160.90 pg/ml (Q2), with a median of 98.40 pg/ml, and the value was higher (p = 0.042) compared with the control group, where it was 35.10 pg/ml [IRQ: 28] (Table 2).

We observed that the MMP-2 concentration was significantly higher in the study group than in the control group (p<0.005, Table 2). The median MMP-2 concentration was 0.76 pg/ml in the experimental subjects, whereas in the control subjects it was 0.41 pg/ml. The median of MMP-9 was 12.3 in the study group and it was lower compared with the control subjects (12.47 pg/ml). The difference was statistically insignificant (p = 0.550, Table 2).

Table 2 also presents Toll like receptor 2 (TLR2) levels in the study group and the control group. Median TLR2 saliva concentration in the experimental group was 0.81 pg/ml, and 1.03 pg/ml in the control group. The difference was not statistically significant (p>0.05).

Correlations between the anthropometric parameters of the women from the study and the control group and the concentrations of cytokines and other proteins were determined in the saliva of the subjects. The results are presented in Tables 3 and 4.

**Table 3:** Correlations (Spearman’s rank correlations) between selected anthropometric parameters and cytokines and other protein levels determined in the saliva of the examined women from the study group.

n = 10	BMI [kg/m^2^]	Waist circumference [cm]
TNF-α [pg/ml]	0.645	0.499
	p = 0.005*	p = 0.041*
Interleukin-8 [pg/ml]	0.494	0.404
	p = 0.044*	p = 0.108
sICAM1/CD24 [pg/ml]	0.350	0.462
	p = 0.169	p = 0.062
Calprotectin [pg/ml]	0.220	0.281
	p = 0.396	p = 0.275
MMP-9 [pg/ml]	-0.189	-0.220
	p = 0.467	p = 0.396
MMP-2 [pg/ml]	0.806	0.796
	p = 0.000*	p = 0.000*
TLR2 [pg/ml]	0.337	0.254
	p = 0.186	p = 0.325

BMI—body mass index, TNF-α–tumor necrosis factor-α, sICAM1/CD24—soluble intercellular adhesion molecule 1, MMP-2 –metalloproteinase-2, MMP-9 –metalloproteinase-9, TLR2 –toll like receptor 2.

**Table 4:** Correlations (Spearman’s rank correlations) between body composition parameters and cytokines and other protein levels determined in the saliva of the examined women from the study group.

n = 10	Fat [kg]	Fat [%]	VAT [cm^3^]	SAT [cm^3^]	VAT/SAT
TNF-α [pg/ml]	0.556	0.548	0.557	0.008	0.624
	p = 0.021*	p = 0.023*	p = 0.020*	p = 0.995	p = 0.007*
Interleukin-8 [pg/ml]	0.535	0.512	0.067	-0.012	0.068
	p = 0.027*	p = 0.036*	p = 0.799	p = 0.963	p = 0.796
sICAM1/CD24 [pg/ml]	0.252	0.218	0.328	0.397	0.423
	p = 0.329	p = 0.402	p = 0.199	p = 0.115	p = 0.091
Calprotectin [pg/ml]	0.208	0.233	0.157	0.549	0.179
	p = 0.422	p = 0.367	p = 0.548	p = 0.022*	p = 0.492
MMP-9 [pg/ml]	-0.144	-0.143	-0.157	-0.338	-0.259
	p = 0.581	p = 0.584	p = 0.548	p = 0.185	p = 0.315
MMP-2 [pg/ml]	0.804	0.794	0.646	0.472	0.701
	p = 0.000*	p = 0.000*	p = 0.005*	p = 0.056	p = 0.002*
TLR2 [pg/ml]	0.230	0.196	0.063	-0.230	0.188
	p = 0.375	p = 0.450	p = 0.810	p = 0.375	p = 0.470

VAT—visceral adipose tissue, SAT—subcutaneous adipose tissue, TNF-α–tumor necrosis factor-α, sICAM1/CD24—soluble intercellular adhesion molecule 1, MMP-2 –metalloproteinase-2, MMP-9 –metalloproteinase-9, TLR2 –toll like receptor 2.

Significant positive correlations of BMI with TNF-α (p = 0.005), IL-8 (p = 0.044), and MMP-2 (p<0.001) were observed. Similarly, the content of fat (in kg and %) correlated positively with TNF-α (p = 0.023), IL-8 (p = 0.036), and MMP-2 (p<0.001). Whereas, the VAT correlated positively only with TNF-α (p = 0.020) and MMP-2 (p = 0.005), similarly to VAT/SAT (ratio of visceral and subcutaneous adipose tissue)–(TNF-α–p = 0.007; MMP-2 –p = 0.002). The WHR was also positively correlated with TNF-α (0 = 0.041) and MMP-2 (p<0.001).

We studied the blood parameters, such as glucose, insulin, HOMA-IR, HDL-cholesterol, LDL-cholesterol, and triglycerides, in the experimental subjects (Table 5) as well as correlations between those parameters and concentrations of the analyzed proteins (Table 6).

**Table 5:** Blood parameter levels (glucose, insulin, HOMA-IR (Homeostatic Model Assessment—Insulin Resistance), HDL-cholesterol, LDL-cholesterol, triglycerides) of the examined individuals from study group.

Parameters:	Study group (n = 10)	
	Median	Q1-Q3
Glucose [mg/dl]	101.00	97.00–105.00
Insulin [mg/dl]	11.15	9.80–12.70
HOMA-IR	48.54	40.51–59.27
HDL-cholesterol [mg/dl]	44.50	43.00–46.00
LDL-cholesterol [mg/dl]	177.00	130.00–204.00
Triglycerides [mg/dl]	169.50	92.00–192.00

HOMA-IR (Homeostatic Model Assessment—Insulin Resistance).

**Table 6:** Correlations (Spearman’s rank correlations) between selected fasting blood parameters and cytokines and other protein levels determined in the saliva of the examined obese women.

n = 10	Glucose [mg/dl]	Insulin [mg/dl]	HOMA-IR	HDL-cholesterol [mg/dl]	LDL-cholesterol [mg/dl]	Triglycerides [mg/dl]
TNF-α [pg/ml]	-0.035	0.657	0.631	-0.183	-0.070	0.407
	p = 0.923	p = 0.039*	p = 0.051	p = 0.614	p = 0.848	p = 0.244
Interleukin-8 [pg/ml]	0.224	-0.278	-0.229	-0.081	-0.137	-0.110
	p = 0.534	p = 0.436	p = 0.524	p = 0.824	p = 0.705	p = 0.763
sICAM1/CD24 [pg/ml]	0.229	0.374	0.419	-0.133	-0.228	0.026
	p = 0.525	p = 0.287	p = 0.228	p = 0.715	p = 0.527	p = 0.944
Calprotectin [pg/ml]	0.131	-0.213	-0.173	0.188	0.167	-0.270
	p = 0.719	p = 0.555	p = 0.634	p = 0.603	p = 0.645	p = 0.451
MMP-9 [pg/ml]	-0.058	0.276	0.252	-0.067	0.238	-0.422
	p = 0.874	p = 0.441	p = 0.483	p = 0.853	p = 0.509	p = 0.224
MMP-2 [pg/ml]	0.360	0.078	0.175	-0.356	-0.117	0.231
	p = 0.307	p = 0.830	p = 0.629	p = 0.313	p = 0.747	p = 0.521
TLR2 [pg/ml]	0.007	0.359	0.337	-0.126	-0.564	0.172
	p = 0.985	p = 0.308	p = 0.341	p = 0.729	p = 0.089	p = 0.634

TNF-α–tumor necrosis factor-α, sICAM1/CD24—soluble intercellular adhesion molecule 1, MMP-2 –metalloproteinase-2, MMP-9 –metalloproteinase-9, TLR2 –toll like receptor 2, HOMA-IR—Homeostatic Model Assessment—Insulin Resistance.

## Discussion

Visceral fat (abdominal adiposity) is associated with the development of adipose cells that are enlarged and dysfunctional. Proinflammatory mediators released by adipose tissue contribute to the development of hyperlipidemia, type 2 diabetes mellitus, or cardiovascular diseases [15].

Different components of blood are secreted into saliva (by passive diffusion, active transport or ultrafiltration between cell junctions). Several blood biomarkers have already been identified in subjects with overweight or obesity. Interestingly, salivary biomarker concentrations were correlated with blood biomarker concentrations, including insulin, cortisol, adipokines, and cytokines [16].

TNF-α enhances insulin resistance, supresseses the expression of adipokine, and increases free fatty acid concentration. In experimental animal models of obesity, TNF-a expression is increased in adipose tissues [17]. In the Lehmann-Kalata study [18], the authors found a statistically significant difference in TNF-a R1 (TNF-a receptor 1) salivary concentration between obese patients and people without obesity (higher in obese subjects), which could indicate an increase in TNF-a concentrations in people with obesity. Similarly, the authors found a statistically significant difference in TNF-a R2 (TNF-a receptor 2) salivary concentration between obese patients and people without obesity (also higher in obese subjects) [18]. In our study, we observed significant positive correlations between BMI with TNF-α (p = 0.005). Similarly, body fat content (in kg and %) correlated positively with TNF-α (p = 0.023).

IL-8 is a cytokine that plays a role in modulating inflammatory response [19]. IL-8 salivary concentrations in our study were correlated with BMI, waist circumference, and fat content. Additionally, IL-8 levels were significantly higher in people from the study group than the control group. Serum concentrations of IL-8 were also significantly higher in obese patients compared with nonobese subjects in the study by Kim et al. [20]. Serum IL-8 levels were also positively related to BMI and waist circumference [20]. In our study, waist circumference did not correlate with IL-8 salivary concentration. Straczkowski et al. concluded that serum IL-8 concentrations were increased in normoglycemic obese subjects and were related to fat mass [19].

Adhesion molecule sICAM-1 levels in the saliva of obese subjects in the Lehmann-Kalata study [21] were statistically higher than those in subjects with normal weight, which could indicate increased risk of leukocyte adhesion to the vascular endothelium [21]. However, in our study, we did not note significant differences in sICAM-1 concentrations between obese and normal weight patients. The increased levels of pro-inflammatory cytokines as well as sICAM1 might also be indicators of atherogenesis, which is connected with chronic processes of inflammation [22].

Calprotectin has been described as a marker of obesity. Catalan et al. [23] showed that serum calprotectin concentrations were increased in both obesity and obesity-related type 2 diabetes mellitus. They noted strong correlations between calprotectin levels and BMI, fat content, waist circumference, and WHR [23]. However, in our study there were no correlations between those anthropometric parameters and salivary calprotectin levels.

Matrix metalloproteinases (MMPs) are synthesized in all tissues and take part in both physiological and pathological processes [24]. Particularly MMP-1, -2 and -9 seem to play a role in the activation or inhibition in tissue remodeling, cardiovascular diseases, and obesity, and they could be related with physical activity [24]. It has been found that long-term resistance training increased MMP-2 and MMP-9, but an acute bout of resistance exercise decreased both MMP-2 and MMP-9 [24]. In our study, we observed significant positive correlations of BMI with MMP-2. Similarly, body fat content (in kg and %) in women correlated positively with MMP-2, however there was no correlation between BMI or fat content and MMP-9.

TLR (Toll-like receptor 2) signaling plays an essential role in obesity as well as metabolic syndrome. In an obese animal model, increased TLR expression was demonstrated. A link between TLR activity and insulin resistance in humans with obesity was also proved. Downregulation of TLR-2 expression leads to biological consequences, such as inhibition of NF-κB signaling, reduced proinflammatory cytokine release, and finally improved body composition [25,26]. We observed increased TLR-2 concentrations in salivary samples from the study group compared with the control subjects, but the difference was not statistically significant.

In our study, we measured the visceral adipose tissue and subcutaneous adipose tissue to analyze their correlation with the concentrations of the analyzed markers. VAT correlated positively only with TNF-α and MMP-2 salivary concentrations, similarly to VAT/SAT (ratio of visceral and subcutaneous adipose tissue). Bruun et al. [27] studied the production and release of IL-8 from subcutaneous and visceral adipose tissues *in vitro*. IL-8 release was four fold higher from VAT than SAT (p<0.05) [27]. In our study, there was no significant correlation between fat area or VAT/SAT ratio and IL-8 concentrations.

The WHR is used to diagnose abdominal obesity and is calculated as a ratio of waist and hip circumferences [2]. In our study, it was also positively correlated with TNF-α (0 = 0.041) and MMP-2 (p<0.001) levels.

Metabolic obesity is more severe in its effects (associated diseases) than metabolic healthy obesity. It is not enough to weigh, measure, determine BMI and WHR to know that this is a metabolic obesity. These indicators are inaccurate and unreliable. In our research, we are looking for quick answers to the question of which patient with obesity should be treated very intensively, e.g. from the beginning using non-pharmacological methods and pharmacotherapy supporting the treatment of obesity (patient diagnosed with metabolic obesity) and to whom we can recommended slow weight reduction with non-pharmacological intervention (diet and increased physical activity).

Our research is a pilot study. Therefore, it was conducted on a small population and a large number of indicator markers form saliva were used, to see if easy to collect material, such as saliva, may be useful for diagnosing early complications of metabolic obesity (insulin resistance) and what markers are worth assessing on a larger population.

## Conclusions

We found that positive correlations of body mass index were observed only with salivary concentrations of TNF-α, MMP-2, and IL-8. Thus, it is worth conducting a study among a larger number of people taking into account these three salivary components.

## Supporting information

S1 File (DOCX)
