# Supplementary material for: Which salivary components can differentiate metabolic obesity?
Source: PLoS One. 2020 Jun 29;15(6):e0235358. doi: 10.1371/journal.pone.0235358 (PMC7323955; doi:10.1371/journal.pone.0235358)
Supplement: S1 File — (DOCX) [file pone.0235358.s001.docx]

| No (study group) | 1 | 2 | 3 | 4 | 5 | 6 | 7 | 8 | 9 | 10 |
| --- | --- | --- | --- | --- | --- | --- | --- | --- | --- | --- |
| Age [years] | 55 | 35 | 48 | 40 | 33 | 61 | 57 | 67 | 49 | 47 |
| Weight [kg] | 90 | 89 | 91 | 84,5 | 78,6 | 91 | 88 | 106 | 94 | 97 |
| Height [m] | 1,69 | 1,67 | 1,63 | 1,58 | 1,52 | 1,64 | 1,67 | 1,65 | 1,58 | 1,64 |
| BMI [kg/m2] | 31,5115 | 31,91222 | 34,25044 | 33,84874 | 34,02008 | 33,83403 | 31,55366 | 38,9348 | 37,65422 | 36,06484 |
| Waist circumference [cm] | 108 | 99 | 108 | 103 | 101 | 112 | 113 | 127 | 114 | 120 |
| Hip circumference [cm] | 118 | 117 | 116 | 117 | 115 | 118 | 106 | 130 | 116 | 123 |
| WHR | 0,915254 | 0,846154 | 0,931034 | 0,880342 | 0,878261 | 0,949153 | 1,066038 | 0,976923 | 0,982759 | 0,97561 |
| Fat adipose tissue [kg] | 38,91 | 34,86 | 40,79 | 37,74 | 30,48 | 43,04 | 32,59 | 50,82 | 45,82 | 46,34 |
| Fat adipose tissue [%] | 43,23 | 39,17 | 44,82 | 44,66 | 38,1 | 47,3 | 38,52 | 47,94 | 48,74 | 47,77 |
| Visceral adipose tissue [cm3] | 117 | 103 | 159 | 102 | 106 | 320 | 350,3 | 359,6 | 359,5 | 729,8 |
| Subcutainous adipose tissue [cm3] | 102 | 87 | 131 | 84 | 83 | 109 | 135 | 104 | 100 | 117 |
| VAT [%] | 53,58 | 54,21 | 54,83 | 54,92 | 56,18 | 74,6 | 75,48 | 77,71 | 78,22 | 86,21 |
| SAT [%] | 46,42 | 45,79 | 45,17 | 45,08 | 43,82 | 25,4 | 24,52 | 22,39 | 21,78 | 13,79 |
| VAT/SAT | 1,15 | 1,18 | 1,21 | 1,22 | 1,28 | 2,94 | 3,08 | 3,47 | 3,59 | 5 |
| TNF-α [pg/ml] | 9,38 | 15,95 | 13,13 | 11,02 | 58,44 | 38,201 | 10,79 | 42,272 | 44,272 | 45 |
| Interleuiin-8 [pg/ml] | 798 | 439 | 451 | 245 | 383 | 231 | 122 | 387 | 368 | 350 |
| sICAM1 CD24 [pg/ml] | 9,24 | 12,8 | 12,8 | 11,6 | 57 | 11,2 | 109 | 56,3 | 44 | 18,12 |
| Calprotectin [pg/ml] | 87,4 | 109,4 | 160,9 | 131 | 47,8 | 163,1 | 384,3 | 55,3 | 40 | 41,4 |
| MMP-9 [pg/ml] | 12,63 | 12 | 12,3 | 12,3 | 12,63 | 12,3 | 11,3 | 12,3 | 10,24 | 12,8 |
| MMP-2 [pg/ml] | 0,875 | 0,32 | 0,7 | 0,65 | 0,72 | 0,68 | 0,9 | 0,95 | 0,8 | 0,85 |
| TLR 2 [pg/ml] | 0,448 | 0,803 | 0,855 | 0,81 | 6,752 | 0,56 | 0,31 | 5,36 | 7,1 | 0,81 |
| Glucose | 101 | 97 | 115 | 86 | 98 | 93 | 105 | 103 | 101 | 105 |
| Insulin | 8,3 | 12 | 8,6 | 10,3 | 18,4 | 9,8 | 12,7 | 12,5 | 10,1 | 16,8 |
| Homa-IR | 37,25778 | 51,73333 | 43,95556 | 39,36889 | 80,14222 | 40,50667 | 59,26667 | 57,22222 | 45,33778 | 78,4 |
| Cholesterol | 187 | 254 | 250 | 163 | 150 | 265 | 221 | 222 | 175 | 275 |
| HDL-cholesterol | 38 | 50 | 63 | 41 | 44 | 46 | 45 | 44 | 45 | 43 |
| LDL-cholesterol | 149 | 204 | 187 | 122 | 106 | 219 | 176 | 178 | 130 | 232 |
| Triglicerides | 146 | 173 | 86 | 92 | 90 | 192 | 174 | 166 | 346 | 355 |

| No (control group) | 1 | 2 | 3 | 4 | 5 | 6 |
| --- | --- | --- | --- | --- | --- | --- |
| Age [years] | 47 | 62 | 32 | 59 | 59 | 28 |
| Weight [kg] | 62,9 | 59,1 | 71,9 | 54,5 | 69,8 | 68 |
| Height [m] | 1,625 | 1,6 | 1,78 | 1,54 | 1,76 | 1,78 |
| BMI [kg/m2] | 23,82012 | 23,08594 | 22,69284 | 22,98027 | 22,53357 | 21,46194 |
| Waist circumference [cm] | 73 | 76,1 | 96 | 80,2 | 95 | 84 |
| Hip circumference [cm] | 97 | 96,1 | 104,8 | 94,7 | 99 | 100 |
| WHR | 0,752577 | 0,791883 | 0,916031 | 0,846885 | 0,959596 | 0,84 |
| Fat adipose tissue [kg] | 17,56 | 17,04 | 21,2 | 16,66 | 17,08 | 16,29 |
| Fat adipose tissue [%] | 27,92 | 28,83 | 29,49 | 30,57 | 24,47 | 23,96 |
| Visceral adipose tissue [cm3] | 54 | 91 | 66 | 124 | 120 | 66 |
| Subcutainous adipose tissue [cm3] | 64 | 93 | 80 | 107 | 110 | 76 |
| VAT [%] | 45,46 | 49,52 | 45,14 | 53,64 | 52,03 | 46,42 |
| SAT [%] | 54,52 | 50,48 | 54,86 | 53,64 | 47,97 | 53,58 |
| VAT/SAT | 0,83 | 0,98 | 0,82 | 1,16 | 1,08 | 0,87 |
| TNF-α [pg/ml] | 16,88 | 15,01 | 15,25 | 11 | 7,2 | 11 |
| Interleuiin-8 [pg/ml] | 397 | 120 | 224 | 125 | 155 | 137 |
| sICAM1 CD24 [pg/ml] | 3,12 | 8,61 | 29,2 | 17,36 | 15,7 | 16,4 |
| Calprotectin [pg/ml] | 17 | 180 | 30,2 | 0 | 40 | 45 |
| MMP-9 [pg/ml] | 12,3 | 12,63 | 12,63 | 12 | 11 | 13 |
| MMP-2 [pg/ml] | 0,5 | 0,45 | 0,35 | 0,4 | 0,3 | 0,42 |
| TLR 2 [pg/ml] | 0,8 | 0 | 4,28 | 0,84 | 1,22 | 1,5 |
